# Diagnostic accuracy of the Timed Up and Go and Five Times Sit-to-Stand tests for fall risk screening in community-dwelling older adults in northern Thailand: a retrospective study

**DOI:** 10.7717/peerj.21041

**Published:** 2026-04-01

**Authors:** Thanatchanok Khieorawong, Arunrat Srithawong

**Affiliations:** 1 Family and Community Medicine Unit, Department of Social Medicine, Chiangrai Prachanukroh Hospital, Chiang Rai, Thailand; 2 Department of Physical Therapy, School of Allied Health Sciences, University of Phayao, Phayao, Thailand

**Keywords:** Aged, Balance, Community-dwelling, Falls, Physical performance

## Abstract

**Background:**

Falls are a major public health concern among older adults, yet simple and context-specific screening tools remain under-validated in Thai settings. This study aimed to evaluate the discriminative performance of the Timed Up and Go Test (TUG) and the Five Times Sit-to-Stand Test (FTSST) for fall risk screening.

**Methods:**

A total of 113 community-dwelling older adults in Chiang Rai, Thailand, were assessed using TUG, FTSST, and the Thai Fall Risk Assessment Tool (Thai-FRAT). Receiver operating characteristic (ROC) curves and logistic regression were used to examine diagnostic accuracy and determine optimal age-specific cut-off values.

**Results:**

The TUG showed good discriminative performance (AUC = 0.827), whereas the FTSST demonstrated more modest discrimination (AUC = 0.705). Each one-second increase in TUG and FTSST times was associated with higher odds of fall risk (TUG: OR = 1.16, 95% CI [1.07–1.27]; FTSST: OR = 1.11, 95% CI [1.02–1.20]). Discriminative performance was higher in adults aged 60–74 years (TUG AUC = 0.871; FTSST AUC = 0.828) than in those aged ≥ 75 years (0.756 and 0.590, respectively). Overall cut-off values were 17.16 seconds for the TUG and 15.46 seconds for the FTSST, with lower thresholds in younger-old adults and higher thresholds in the oldest-old.

**Conclusion:**

The TUG demonstrated robust discriminative performance for community-based fall risk screening, whereas the FTSST may be more suitable as a complementary tool. Incorporating age-specific cut-off values may enhance interpretation and support early identification and prevention of falls among older adults.

## Introduction

Thailand is undergoing a significant demographic shift, with projections indicating that more than 30% of the population will be aged 60 years and older by 2030 (Anantanasuwong, 2021). This rapid ageing trend presents pressing public health challenges, particularly in the prevention of falls, which are a major contributor to morbidity, disability, and loss of independence among older adults (Giovannini et al., 2022). Falls are associated with a wide range of adverse health outcomes, including fractures, traumatic brain injuries, and increased risk of institutionalization and mortality (Berg & Cassells, 1992; Larsson et al., 2019). Furthermore, the economic burden of fall-related injuries is considerable, encompassing both direct medical expenses and indirect costs such as caregiver burden and reduced quality of life (Florence et al., 2018; Watson, Clapperton & Mitchell, 2011).

In the Thai context, national surveillance data indicate an increasing trend in fall-related hospitalizations and deaths among older adults. In Chiang Rai province, the fall-related mortality rate among older adults increased from 15.8 to 24.36 deaths per 100,000 older adults between 2017 and 2023 (Department of Disease Control MoPH, 2022). Early identification of individuals at elevated risk of falling is therefore a critical public health priority to enable the timely implementation of evidence-based interventions. Targeted strategies such as strength and balance training and home environment modification have demonstrated efficacy in reducing fall incidence among older populations (Lee & Yu, 2020; Sherrington et al., 2019).

Accordingly, practical and effective screening approaches are needed in community settings. Functional performance tests are widely recommended for fall-risk screening because they are low-cost, easy to administer, and feasible for large-scale use among community-dwelling older adults (Barry et al., 2014; Ong et al., 2023). These tools support early identification of individuals at increased risk and facilitate timely referral to targeted prevention strategies, including strength and balance training, environmental modification, and multifactorial interventions such as comprehensive geriatric assessment (Lee & Yu, 2020; Montero-Odasso et al., 2022). The Five Times Sit-to-Stand Test (FTSST) and the Timed Up and Go Test (TUG) are frequently utilized. The FTSST assesses lower limb strength and balance by measuring the time required to complete five sit-to-stand repetitions (Park & Shin, 2024), while the TUG evaluates dynamic balance and mobility through a timed sequence of standing, walking, turning, and sitting (Herman, Giladi & Hausdorff, 2011). Both tests have demonstrated strong inter-rater reliability and are responsive to change following interventions (Sancar, Doğan & Taş, 2024). Although both tests have demonstrated strong inter rater reliability and responsiveness to intervention their ability to accurately identify individuals at risk of falling as reflected by sensitivity specificity and optimal cut off values vary across populations (Sancar, Doğan & Taş, 2024). Moreover, evidence supporting their effectiveness for fall risk screening among older adults living in rural or semi urban Thai settings remains limited.

The validity and feasibility of fall risk assessments such as the TUG and the FTSST may be influenced by contextual factors, including physical infrastructure, service accessibility, and cultural perceptions of aging (Lektip et al., 2021; Trongsakul & Vimolratana, 2018). Although these tools are widely applied in geriatric practice worldwide, comparative validation in Thai older adults remains limited, particularly in semi-urban or rural provinces such as Chiang Rai. This gap highlights the need for context-specific and clinically meaningful cut-off values that can guide routine fall risk screening and inform preventive strategies in Thailand. Therefore, the present study aimed to assess and compare the diagnostic discriminative accuracy of the TUG and the FTSST for identifying fall risk among community-dwelling older adults in Chiang Rai, and to establish age-specific cut-off values for both tests.

## Materials & Methods

### Study design and participants

This study employed a cross-sectional design using retrospectively collected data from electronic medical records at the Chiangrai Prachanukroh Hospital, covering the period from January 2022 to December 2024. Electronic medical records consisted of routinely collected digital clinical data maintained as part of standard care in the rehabilitation unit, including demographic and clinical information as well as standardized physical performance assessments conducted by licensed physical therapists. The study was approved by the Institutional Review Board of Chiangrai Prachanukroh Hospital (EC CRH 065/68 In) and conducted in accordance with the Declaration of Helsinki. Therefore, only secondary data were analyzed without direct participant contact, the requirement for informed consent was waived.

Eligible participants included community-dwelling adults aged 60 years and older residing in Rop Wiang Subdistrict, Mueang Chiang Rai District, Chiang Rai Province, which lies within the hospital’s service catchment area. All individuals had undergone routine fall risk screening conducted by licensed physical therapists and had complete data from two standardized assessments: the FTSST, and the TUG. Inclusion criteria were: (1) age ≥ 60 years, (2) residence within the hospital’s service catchment area; and (3) availability of complete data on demographic characteristics, anthropometric measures, medical comorbidities, Thai-FRAT score, fall history, and use of ambulatory assistive devices. Exclusion criteria included: (1) dependence on a wheelchair for mobility, (2) diagnosis of severe neuromuscular or musculoskeletal disorders such as Parkinson’s disease, stroke, or advanced osteoarthritis, and (3) moderate to severe cognitive impairment as judged by clinical documentation.

### Data collection

Baseline demographic, anthropometric, and clinical information were extracted from electronic medical records, including age, sex, body mass index, comorbidity profiles, use of ambulatory assistive devices, and fall history within the preceding six months. All data were obtained from routinely collected clinical records documented by licensed healthcare professionals. Fall risk was classified using the Thai-FRAT, a validated screening instrument developed for older adults in Thailand. The Thai-FRAT comprises six established fall-risk factors: sex, visual acuity, balance ability, medication use, fall history, and housing characteristics. Items are weighted according to the original scoring system, yielding a total score ranging from 0 to 11, with higher scores indicating greater fall risk. Participants with a Thai-FRAT score ≥ 4 were classified as fall-risk (Thiamwong et al., 2008). In addition, individuals with a documented history of at least one fall within the preceding six months were also classified as fall-risk regardless of their Thai-FRAT score. Participants who met neither criterion were classified as non-risk.

### Physical performance assessments

All participants underwent two standardized physical performance tests under the supervision of licensed physical therapists in a controlled clinical environment. During testing, participants wore their usual footwear and were allowed to use their customary ambulatory assistive devices as needed for safe ambulation. These devices included walkers, tripod canes, and single-point canes, and their use reflected participants’ habitual daily ambulation as documented in the electronic medical records (Table 1). A lightweight safety harness was used to minimize risk. One practice trial was performed, followed by two recorded trials. The mean of the two recorded trials was used for analysis, and performance time was recorded in seconds (Steffen, Hacker & Mollinger, 2002). Participants were allowed to rest in a seated position for approximately 1–2 min between trials to minimize fatigue (Sancar, Doğan & Taş, 2024).

### FTSST

The FTSST was used to assess lower extremity muscle strength. Participants were seated on a standard armless chair with a seat height of 43 cm, with feet positioned comfortably on the floor. After standardized verbal instructions and a brief demonstration, participants were instructed to stand up and sit down five times as quickly as possible at their maximal safe speed, ensuring full hip and knee extension during each standing phase. Participants were instructed not to use their upper limbs to assist with rising from the chair unless required for safety. Timing commenced at the verbal command “go” and ended when the participant completed the fifth sit-down. Contact with the chair backrest was permitted only at the completion of the final repetition. Minor foot adjustments were allowed as needed to maintain balance and safety (Makizako et al., 2017; Whitney et al., 2005).

**Table 1:** Baseline characteristics and physical performance measures of the study participants. Demographic characteristics and physical performance measures of the participants. Data are summarized as means with standard deviations or as counts with percentages, as appropriate.

**Variables**	**Non-risk (*n* = 52)**	**Fall-risk (*n* = 61)**	***p*-value**
Age (years), mean ± SD	75.29 ± 7.95	76.89 ± 7.19	0.265
BMI (kg/m^2^), mean ± SD	23.86 ± 4.67	23.35 ± 4.93	0.572
Sex, n (%)			
Male	7.00 (13.50)	19.00 (31.10)	0.026^*^
Female	45.00 (86.50)	42.00 (68.90)	
Comorbidities, n (%)			
None	1.00 (1.90)	4.00 (6.60)	
Metabolic only^a^	0.00 (0.00)	2.00 (3.30)	0.235
Cardiovascular only^b^	5.00 (9.60)	9.00 (14.80)	
Multiple systems^c^	46.00 (88.50)	46.00 (75.40)	
Thai-FRAT score, mean ± SD	3.80 ± 0.4	8.90 ± 3.20	<0.001^*^
Fall frequency, n (%)			
0 time	52.00 (100.00)	42.00 (68.90)	<0.001^*^
≥1 times	0.00 (0.00)	19.00 (31.10)	
Use of gait aid, n (%)			
None	48.00 (92.30)	21.00 (34.40)	
Walker	0.00 (0.00)	11.00 (18.00)	<0.001^*^
Tripod cane	4.00 (7.70)	11.00 (18.00)	
Single-point cane	0.00 (0.00)	18.00 (29.50)	
TUG (seconds), mean ± SD	14.80 ± 6.10	25.40 ± 11.60	<0.001^*^
FTSST (seconds), mean ± SD	15.30 ± 5.90	18.90 ± 5.90	0.002^*^

**Notes.**

Continuous variables are presented as mean ± SD; categorical variables as number (percentage). TUG, Timed up and go test; FTSST, Five times sit to stand test; Thai-FRAT, Thai Falls Risk Assessment Test.

* *p* < 0.05 indicates statistical significance.

a Metabolic: diabetes mellitus, dyslipidemia, or gout.

b Cardiovascular: hypertension, coronary artery disease, or atrial fibrillation.

c Multiple systems: ≥2 comorbidities across different system.

### TUG

The TUG test was used to assess functional mobility and dynamic balance in older adults. A three-meter walkway was marked on the floor in front of a standard armrest chair, with a cone placed at the distal end serving as the turning point. Participants began the test in a seated position with their back supported by the chair backrest, arms resting on their thighs, and feet positioned just behind the starting line. On a standardized verbal command, participants stood up from the chair, walked at a comfortable but safe pace to the cone, turned around it, walked back to the chair, and sat down fully. Participants were informed in advance that the task would be timed. Timing commenced at the verbal start command and ended when the participant returned to the seated position with their back contacting the chair backrest (Lusardi, Pellecchia & Schulman, 2003; Steffen, Hacker & Mollinger, 2002).

### Statistics

Descriptive statistics were used to summarize participant characteristics. Continuous variables were expressed as mean ± standard deviation (SD), while categorical variables were presented as frequencies and percentages. The Shapiro–Wilk test was applied to assess the normality of continuous data. Between-group comparisons were performed using the independent samples *t*-test for normally distributed variables and the Mann–Whitney U test for non-normally distributed variables. Categorical variables (*e.g.*, sex, comorbidity profile, fall history, and use of assistive devices) were compared using the Chi-square test. Binary logistic regression was conducted to examine the associations of TUG and FTSST with fall-risk status, adjusting for potential confounders (age, sex, and use of gait aid). Odds ratios (ORs) with 95% confidence intervals (CIs) were calculated to assess associations between functional performance measures and fall risk. Model fit was evaluated using pseudo R^2^, and multicollinearity among independent variables was examined using variance inflation factors (VIFs). Receiver operating characteristic (ROC) curve analysis was performed to assess discriminative performance and to determine optimal cut-off values for the TUG and the FTSST using Youden’s index, with corresponding sensitivity, specificity, and AUC values with 95% confidence intervals reported. Pearson’s correlation analysis was used to examine the relationships among TUG, FTSST, and Thai-FRAT scores. All statistical analyses were performed using Stata version 14.0 (StataCorp, College Station, TX, USA). A two-sided *p*-value < 0.05 was considered statistically significant.

## Results

The baseline characteristics of participants are presented in Table 1. Among the 113 older adults, 61 (53.9%) were classified as fall-risk and 52 (46.1%) as non-risk. There were no significant differences in age, body mass index, or comorbidity profiles between groups (*p* > 0.05). In contrast, sex distribution, Thai-FRAT scores, gait aid use, and functional performance differed significantly. A higher proportion of males and greater reliance on gait aids were observed in the fall-risk group. Moreover, participants at risk demonstrated significantly longer completion times on both the TUG and FTSST, as well as markedly higher Thai-FRAT scores (*p* < 0.05).

In adjusted logistic regression analyses (Table 2), both the TUG and FTSST were independently associated with fall-risk status after adjustment for age, sex, and use of ambulatory aids. Each one-second increase in TUG time was associated with higher odds of fall risk (OR = 1.16, 95% CI [1.07–1.27]; *p* < 0.001), as was each one-second increase in FTSST time (OR = 1.11, 95% CI [1.02–1.20]; *p* = 0.013). The pseudo-R^2^ values were 0.40 for the TUG model and 0.32 for the FTSST model. All variance inflation factors were < 2 (mean VIF = 1.40).

**Table 2:** Logistic regression estimates for the association between Timed Up and Go and Five Times Sit-to-Stand performance and fall-risk status. Logistic regression models were used to estimate associations between test performance and fall-risk classification. Results are reported as odds ratios with 95% confidence intervals, with models adjusted for prespecified covariates.

**Tests**	**OR (95% CI)**	***p*-value**
TUG (per seconds)	1.16 (1.07–1.27)	<0.001^*^
FTSST (per seconds)	1.11 (1.02–1.20)	0.013^*^

**Notes.**

OR odds ratio CI confidence interval

Adjusted for age, sex, and use of gait aid.

* *p* < 0.05 indicates statistical significance.

**Figure 1:**
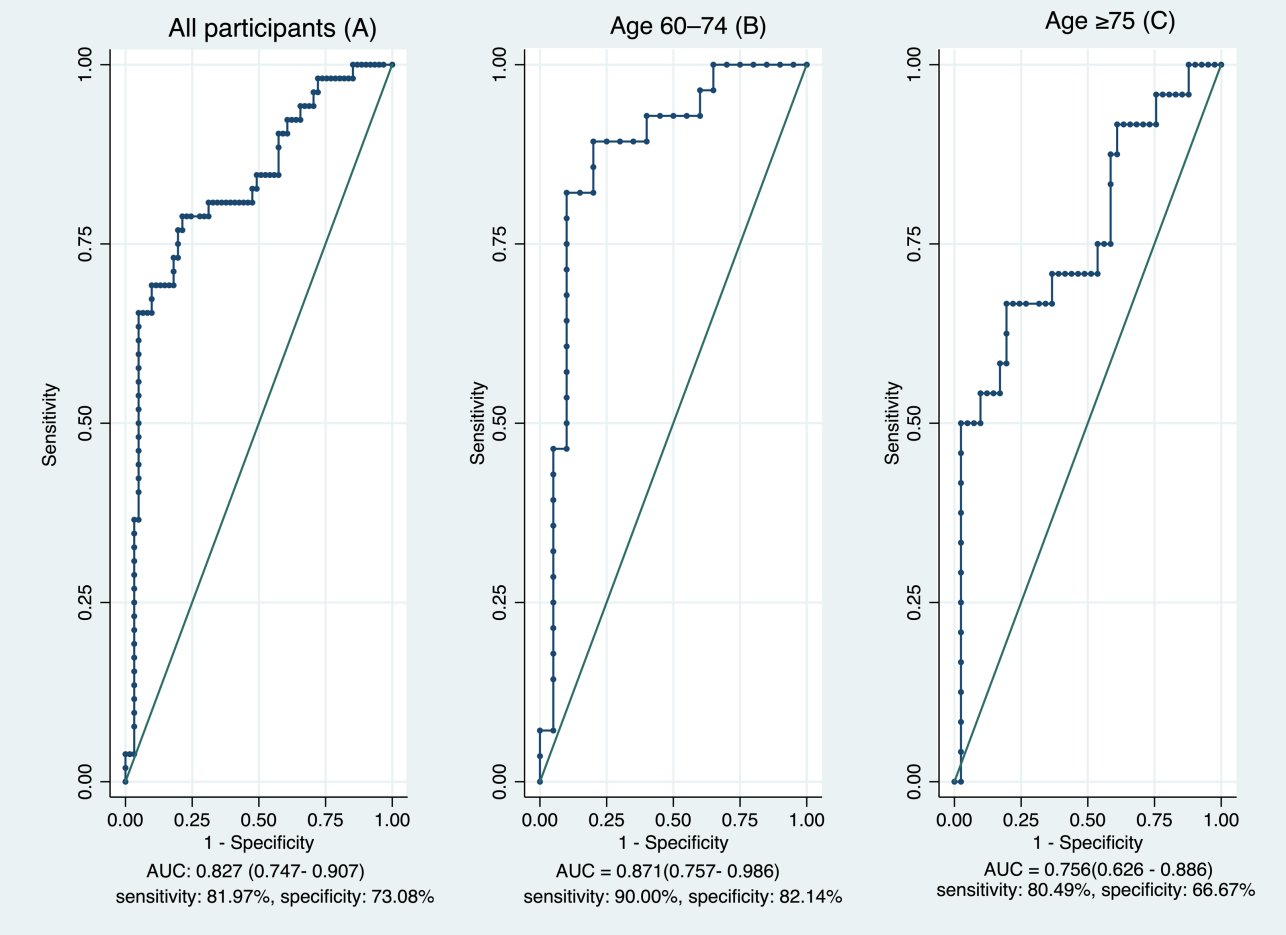
Discriminative performance of the Timed Up and Go Test for fall-risk identification across age groups. Receiver operating characteristic curves for the Timed Up and Go Test in the overall sample (A) and stratified by age group: 60–74 years (B) and ≥75 years (C).

**Figure 2:**
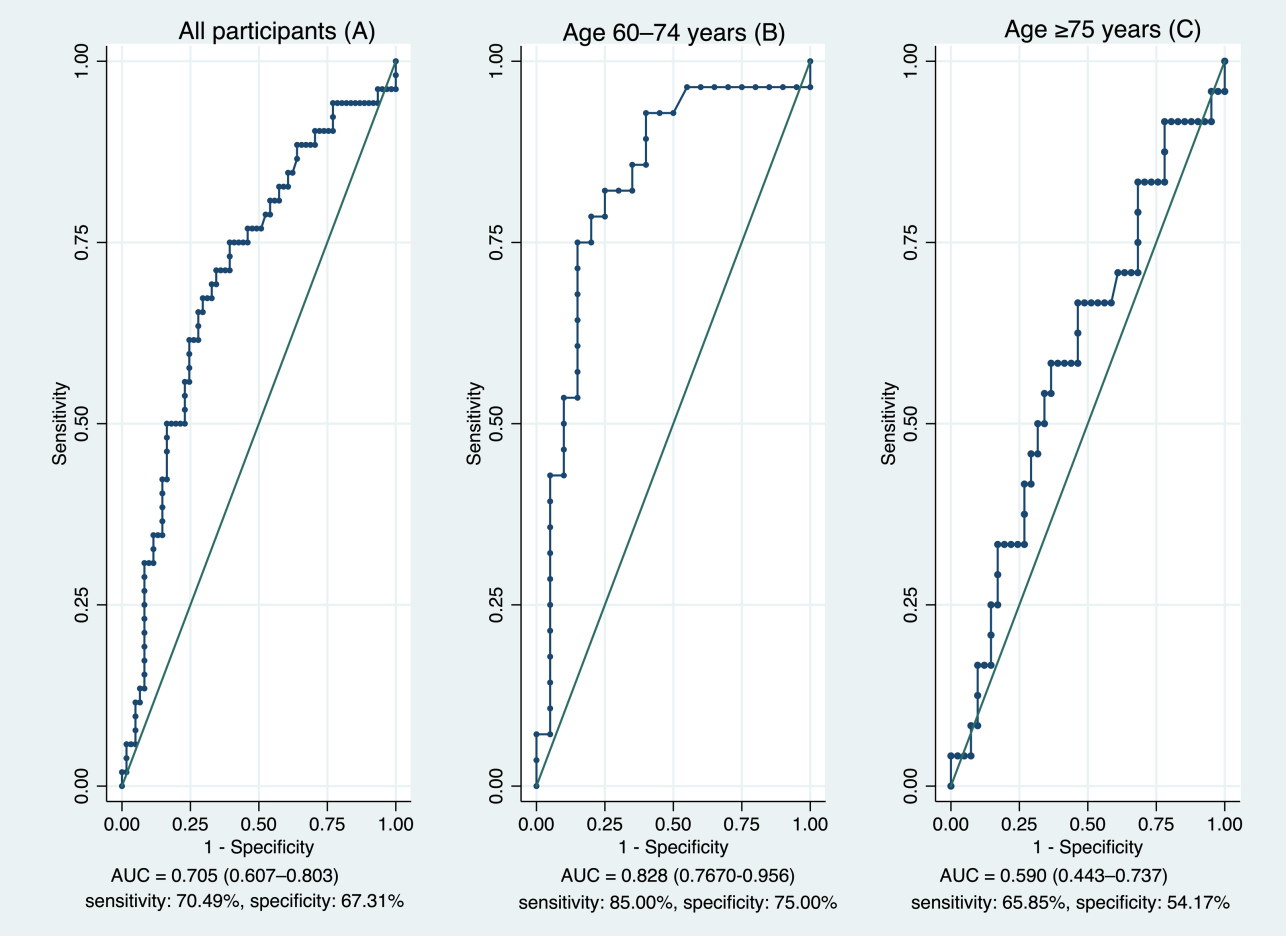
Discriminative performance of the Five Times Sit-to-Stand Test for fall-risk identification across age groups. Receiver operating characteristic curves for the Five Times Sit-to-Stand Test in the overall sample (A) and stratified by age group: 60–74 years (B) and ≥75 years (C).

As shown in Table 3, ROC analyses indicated that both the TUG and FTSST discriminated between fall-risk and non-risk participants (Table 3). In the total sample, the optimal cut-off for the TUG was 17.16 s (AUC = 0.827, 95% CI [0.747–0.907]; sensitivity 81.97%, specificity 73.08%) (Fig. 1A), whereas the corresponding cut-off for the FTSST was 15.46 s (AUC = 0.705, 95% CI [0.607–0.803]; sensitivity 70.49%, specificity 67.31%) (Fig. 2A). Age-stratified analyses showed higher discriminative performance in participants aged 60–74 years than in those aged ≥75 years. In the 60–74-year group, the TUG and FTSST demonstrated AUCs of 0.871 (95% CI [0.757–0.986]) and 0.828 (95% CI [0.767–0.956]), respectively (Figs. 1B and 2B). In contrast, among participants aged ≥75 years, AUC values declined to 0.756 for the TUG (95% CI [0.626–0.886]) and 0.590 for the FTSST (95% CI [0.443–0.737]) (Figs. 1C and 2C). Correlation coefficients between functional tests and Thai-FRAT scores are summarized in Table 4. Both TUG and FTSST were significantly correlated with Thai-FRAT scores (*r* = 0.406 and *r* = 0.319, respectively; *p* < 0.001). A strong correlation was also observed between TUG and FTSST (*r* = 0.646, *p* < 0.001).

**Table 3:** Cut-off values and diagnostic accuracy of the Timed Up and Go and Five Times Sit-to-Stand tests for fall-risk identification. Cut-off values were derived from receiver operating characteristic analyses. Diagnostic accuracy is summarized using the area under the curve, sensitivity, and specificity.

**Tests**	**Age group**	**n**	**Cut-off** **(seconds)**	**Sensitivity (%)**	**Specificity** **(%)**	**AUC** **(95% CI)**
TUG	All participants	113	17.16	81.97	73.08	0.827 (0.747–0.907)
	60–74 years	48	15.74	90.00	82.14	0.871 (0.757–0.986)
	≥75 years	65	17.43	80.49	66.67	0.756 (0.626–0.886)
FTSST	All participants	113	15.46	70.49	67.31	0.705 (0.607–0.803)
	60–74 years	48	14.58	85.00	75.00	0.828 (0.767–0.956)
	≥75 years	65	15.50	65.85	54.17	0.590 (0.443–0.737)

**Notes.**

TUG Timed up and go test FTSST Five times sit to stand test AUC Area under the curve CI Confidence interval

**Table 4:** Correlation coefficients among Timed Up and Go, Five Times Sit-to-Stand, and Thai Fall Risk Assessment Test scores. Correlation coefficients describe the direction and magnitude of associations between functional performance tests and fall-risk assessment scores.

**Variables**	**Thai-FRAT**	**TUG**
Thai-FRAT (scores)	1.000	0.406^*^
TUG (seconds)	0.406^*^	1.000
FTSST (seconds)	0.319^*^	0.646^*^

**Notes.**

TUG Timed Up and Go Test FTSST Five Times Sit-to-Stand Test Thai-FRAT Thai Falls Risk Assessment Tool

* *p* < 0.05 indicates statistical significance.

## Discussion

This study investigated the discriminative performance of the TUG and the FTSST for identifying fall risk among community-dwelling older adults. Both tests demonstrated significant discriminative capacity; however, their accuracy differed across age strata, indicating that age-stratified interpretation may be necessary when these tools are used for clinical screening.

These results indicate that the TUG demonstrated higher discriminative performance (AUC = 0.827), whereas the FTSST showed more modest discrimination (AUC = 0.705) (Çorbacıoğlu & Aksel, 2023). After adjustment for relevant covariates, poorer performance on both tests remained independently associated with higher fall-risk status, with a more stable association observed for the TUG across models. Taken together, these findings support the use of the TUG as a primary performance-based screening measure, while the FTSST may be more appropriately interpreted as a complementary assessment rather than a stand-alone tool.

The observed differences in discriminative performance between the TUG and the FTSST likely reflect the distinct functional domains assessed by each test. The TUG involves multiple sequential tasks, including rising from a chair, walking, turning, and sitting, and therefore requires the integration of lower extremity strength, dynamic balance, gait control, and cognitive sequencing (Herman, Giladi & Hausdorff, 2011; Nocera et al., 2013). As a result, the TUG captures multidimensional mobility demands that closely resemble those encountered during daily activities in which falls commonly occur (Walz et al., 2023). In contrast, the FTSST primarily assesses lower limb muscle strength and power, particularly quadriceps function, during repeated sit-to-stand movements (Van Lummel et al., 2018). While this capacity is important for functional independence, the FTSST places less emphasis on balance control, gait, and transitional movements, which are recognized as critical components of fall risk in older adults (Barry et al., 2014; Shumway-Cook, Brauer & Woollacott, 2000; Tinetti & Kumar, 2010). Previous studies have reported moderate correlations between the TUG and the FTSST, indicating that the two tests share common underlying components related to lower extremity function while assessing partially distinct aspects of physical performance (Makizako et al., 2017). Consistent with these findings, the moderate intercorrelation observed in the present study (*r* = 0.646) suggests overlapping yet distinct functional constructs. Together, these differences may explain the greater discriminative performance of the TUG for fall-risk identification compared with the more strength-focused FTSST.

Notably, both performance-based measures demonstrated only weak correlations with Thai-FRAT scores (*r* = 0.406 and *r* = 0.319, respectively), substantiating the interpretation that objective functional assessments and multifactorial risk stratification instruments capture complementary rather than redundant dimensions of fall risk. This discordance likely reflects the Thai-FRAT’s emphasis on static balance evaluation and incorporation of diverse risk factors including medical comorbidities, medication profiles, and historical fall events (Thiamwong et al., 2008), which are not directly captured through timed performance measures. These findings highlight the importance of comprehensive fall-risk assessment that integrates both objective performance measures and multifactorial screening tools.

Age-stratified analyses revealed clinically meaningful differences in test performance across age cohorts. In participants aged 60–74 years, both the TUG and FTSST demonstrated good discriminative ability, with AUC values of 0.871 and 0.828, respectively. These findings suggest that, in this younger-old group, performance-based measures are able to more clearly distinguish between fall-risk categories, likely reflecting relatively preserved functional capacity and less heterogeneity in physical performance.

In contrast, discriminative performance declined among participants aged ≥75 years, particularly for the FTSST, which demonstrated poor discriminative capability (AUC = 0.590) (Çorbacıoğlu & Aksel, 2023). Although the TUG retained moderate discriminative performance in the oldest-old (AUC = 0.756), its accuracy was also reduced compared with younger participants. These findings indicate that the ability of performance-based tests to discriminate fall-risk status diminishes with advancing age, with the decline being more pronounced for strength-focused measures such as the FTSST.

The reduced discriminative accuracy observed in adults aged ≥75 years likely reflects increasing heterogeneity in physical function and health status with advancing age. In this age group, wider variability in mobility, frailty, and comorbidity burden may limit the ability of single performance-based tests to clearly distinguish fall-risk categories (Chung et al., 2023; Fried et al., 2001). Age-related neuromuscular changes, including declines in muscle strength, coordination, reaction time, and postural control, have been shown to increase variability in functional performance among older adults (Hsu, Chou & Woollacott, 2013; Hunter, Pereira & Keenan, 2016), thereby narrowing the discriminative range of tests that assess isolated functional domains. In addition, because fall history formed part of the reference standard in this cross-sectional analysis, performance on mobility-based tests may partly reflect the functional consequences of previous falls rather than exclusively indicating future risk. Despite this limitation, the relatively preserved discriminative performance of the TUG across age groups suggests greater applicability of this measure in more advanced age. Accordingly, the present findings should be interpreted as reflecting the ability of the TUG and FTSST to discriminate fall-risk status at a single point in time rather than to predict incident falls, highlighting the need for prospective studies that longitudinally capture fall events, particularly in the oldest-old.

The overall cut-off values identified in the present study (17.16 s for the TUG and 15.46 s for the FTSST) fall within ranges reported in previous studies, although substantial variability across populations and age groups has been documented (Buatois et al., 2008; Kang et al., 2017; Shumway-Cook, Brauer & Woollacott, 2000; Wada et al., 2023). Normative data from Thai older adults, reporting mean values of 11.0 ± 2.4 s for the TUG and 14.1 ± 3.2 s for the FTSST (Suwannarat et al., 2021; Thaweewannakij et al., 2013), further contextualize these findings. The higher thresholds observed in individuals classified as fall-risk likely reflect functional impairment beyond typical age-related decline, supporting the clinical relevance of these measures for fall-risk discrimination when interpreted with consideration of age-related functional heterogeneity.

Several limitations warrant consideration when interpreting these findings. First, recruitment from a single geographic region may limit the generalizability of the results. Second, the use of fall history and Thai-FRAT as reference standards does not capture all dimensions of fall risk, such as medication use, visual impairment, environmental hazards, and fear of falling. In addition, the lower discriminative performance observed in adults aged ≥75 years likely reflects greater heterogeneity in physical function, frailty, and comorbidity burden in this age group. Because fall history was assessed retrospectively, test performance may partly reflect the consequences of prior falls rather than future risk. Therefore, prospective studies using incident falls as the primary outcome, with larger and more diverse samples, are warranted to validate these findings and to further refine the role of the TUG and FTSST in fall-risk assessment.

## Conclusions

The TUG demonstrated higher discriminative performance than the FTSST for fall-risk screening among community-dwelling older adults, whereas the FTSST showed more limited discrimination, particularly in adults aged ≥75 years. These findings support the use of the TUG as a primary performance-based screening measure, with the FTSST serving as a complementary test within a multifactorial fall-risk assessment framework. Population- and age-specific cut-off values may enhance interpretability; however, external validation is required prior to widespread clinical implementation.

##  Supplemental Information

10.7717/peerj.21041/supp-1 Supplemental Information 1 STROBE Checklist for Cross-sectional Study

10.7717/peerj.21041/supp-2 Supplemental Information 2 Raw data

## Additional Information and Declarations

### Data Availability

The following information was supplied regarding data availability: The raw measurements of participant demographics, clinical characteristics, and functional test results (TUG, FTSST, *etc.*) are available in the Supplemental File.

## References

[ref-1] Anantanasuwong D, Narot P, Kiettikunwong N (2021). Population ageing in Thailand: critical issues in the twenty-first century. Education for the elderly in the Asia-Pacific region: issues, concerns and prospects.

[ref-2] Barry E, Galvin R, Keogh C, Horgan F, Fahey T (2014). Is the timed up and go test a useful predictor of risk of falls in community dwelling older adults: a systematic review and meta- analysis. BMC Geriatrics.

[ref-3] Berg RL, Cassells JS, Berg RL, Cassells JS (1992). Falls in older persons: risk factors and prevention. The second fifty years: promoting health and preventing disability.

[ref-4] Buatois S, Miljkovic D, Manckoundia P, Gueguen R, Miget P, Vançon G, Perrin P, Benetos A (2008). Five times sit to stand test is a predictor of recurrent falls in healthy community-living subjects aged 65 and older. Journal of the American Geriatrics Society.

[ref-5] Çorbacıoğlu ŞK, Aksel G (2023). Receiver operating characteristic curve analysis in diagnostic accuracy studies: a guide to interpreting the area under the curve value. Turkish Journal of Emergency Medicine.

[ref-6] Chung E, Lee S-H, Lee H-J, Kim Y-H (2023). Comparative study of young-old and old-old people using functional evaluation, gait characteristics, and cardiopulmonary metabolic energy consumption. BMC Geriatrics.

[ref-7] Department of Disease Control MoPH (2022). Falls among older adults aged 60 and above in Thailand. https://ddc.moph.go.th/dip/news.php?news=23567&deptcode=.

[ref-8] Florence CS, Bergen G, Atherly A, Burns E, Stevens J, Drake C (2018). Medical costs of fatal and nonfatal falls in older adults. Journal of the American Geriatrics Society.

[ref-9] Fried LP, Tangen CM, Walston J, Newman AB, Hirsch C, Gottdiener J, Seeman T, Tracy R, Kop WJ, Burke G, McBurnie MA (2001). Frailty in older adults: evidence for a phenotype. The Journals of Gerontology. Series A, Biological Sciences and Medical Sciences.

[ref-10] Giovannini S, Brau F, Galluzzo V, Santagada DA, Loreti C, Biscotti L, Laudisio A, Zuccalà G, Bernabei R (2022). Falls among older adults: screening, identification, rehabilitation, and management. Applied Sciences.

[ref-11] Herman T, Giladi N, Hausdorff JM (2011). Properties of the ‘timed up and go’ test: more than meets the eye. Gerontology.

[ref-12] Hsu W-L, Chou L-S, Woollacott M (2013). Age-related changes in joint coordination during balance recovery. Age.

[ref-13] Hunter SK, Pereira HM, Keenan KG (2016). The aging neuromuscular system and motor performance. Journal of Applied Physiology.

[ref-14] Kang L, Han P, Wang J, Ma Y, Jia L, Fu L, Hairui Y, Chen X, Niu K, Guo Q (2017). Timed up and go test can predict recurrent falls: a longitudinal study of the community-dwelling elderly in China. Clinical Interventions in Aging.

[ref-15] Larsson L, Degens H, Li M, Salviati L, Lee YI, Thompson W, Kirkland JL, Sandri M (2019). Sarcopenia: aging-related loss of muscle mass and function. Physiological Reviews.

[ref-16] Lee SH, Yu S (2020). Effectiveness of multifactorial interventions in preventing falls among older adults in the community: a systematic review and meta-analysis. International Journal of Nursing Studies.

[ref-17] Lektip C, Lapmanee S, Rattananupong T, Lohsoonthorn V, Vorayingyong A, Woratanarat T, Sirisuk K-O, Suttanon P, Petsirasan R, Kitidumrongsuk P, Jiamjarasrangsi W (2021). Predictive validity of three home fall hazard assessment tools for older adults in Thailand. PLOS ONE.

[ref-18] Lusardi MM, Pellecchia GL, Schulman M (2003). Functional performance in community living older adults. Journal of Geriatric Physical Therapy.

[ref-19] Makizako H, Shimada H, Doi T, Tsutsumimoto K, Nakakubo S, Hotta R, Suzuki T (2017). Predictive cutoff values of the five-times sit-to-stand test and the timed up & go test for disability incidence in older people dwelling in the community. Physical Therapy.

[ref-20] Montero-Odasso M, Van der Velde N, Martin FC, Petrovic M, Tan MP, Ryg J, Aguilar-Navarro S, Alexander NB, Becker C, Blain H, Bourke R, Cameron ID, Camicioli R, Clemson L, Close J, Delbaere K, Duan L, Duque G, Dyer SM, Freiberger E, Ganz DA, Gómez F, Hausdorff JM, Hogan DB, Hunter SMW, Jauregui JR, Kamkar N, Kenny RA, Lamb SE, Latham NK, Lipsitz LA, Liu-Ambrose T, Logan P, Lord SR, Mallet L, Marsh D, Milisen K, Moctezuma-Gallegos R, Morris ME, Nieuwboer A, Perracini MR, Pieruccini-Faria F, Pighills A, Said C, Sejdic E, Sherrington C, Skelton DA, Dsouza S, Speechley M, Stark S, Todd C, Troen BR, Van der Cammen T, Verghese J, Vlaeyen E, Watt JA, Masud T (2022). World guidelines for falls prevention and management for older adults: a global initiative. Age and Ageing.

[ref-21] Nocera JR, Stegemöller EL, Malaty IA, Okun MS, Marsiske M, Hass CJ (2013). Using the timed up & go test in a clinical setting to predict falling in Parkinson’s disease. Archives of Physical Medicine and Rehabilitation.

[ref-22] Ong MF, Soh KL, Saimon R, Myint WW, Pawi S, Saidi HI (2023). Falls risk screening tools intended to reduce fall risk among independent community-dwelling older adults: a systematic review. International Journal of Nursing Practice.

[ref-23] Park TS, Shin MJ (2024). Comprehensive assessment of lower limb function and muscle strength in sarcopenia: insights from the Sit-to-Stand Test. Annals of Geriatric Medicine and Research.

[ref-24] Sancar B, Doğan A, Taş S (2024). Timed up-and-go test and sit-to-stand test in community-dwelling older person: inter and intraobserver reliability among turkish nurses. International Journal of Older People Nursing.

[ref-25] Sherrington C, Fairhall NJ, Wallbank GK, Tiedemann A, Michaleff ZA, Howard K, Clemson L, Hopewell S, Lamb SE (2019). Exercise for preventing falls in older people living in the community. Cochrane Database of Systematic Reviews.

[ref-26] Shumway-Cook A, Brauer S, Woollacott M (2000). Predicting the probability for falls in community-dwelling older adults using the Timed Up & Go Test. Physical Therapy.

[ref-27] Steffen TM, Hacker TA, Mollinger L (2002). Age- and gender-related test performance in community-dwelling elderly people: six-Minute Walk Test, Berg Balance Scale, Timed Up & Go Test, and gait speeds. Physical Therapy.

[ref-28] Suwannarat P, Kaewsanmung S, Thaweewannakij T, Amatachaya S (2021). The use of functional performance tests by primary health-care providers to determine walking ability with and without awalking device in community-dwelling elderly. Physiotherapy Theory and Practice.

[ref-29] Thaweewannakij T, Wilaichit S, Chuchot R, Yuenyong Y, Saengsuwan J, Siritaratiwat W, Amatachaya S (2013). Reference values of physical performance in thai elderly people who are functioning well and dwelling in the community. Physical Therapy.

[ref-30] Thiamwong L, Thamarpirat J, Maneesriwongul W, Jitapunkul S (2008). Thai falls risk assessment test (Thai-FRAT) developed for community-dwelling Thai elderly. Journal of the Medical Association of Thailand.

[ref-31] Tinetti ME, Kumar C (2010). The patient who falls: “It’s always a trade-off”. JAMA.

[ref-32] Trongsakul S, Vimolratana O (2018). Prevalence and associated factors of fall risk in Thai older people: a primary care based study in Chiang Rai. Journal of Current Science and Technology.

[ref-33] Van Lummel RC, Evers J, Niessen M, Beek PJ, Van Dieën JH (2018). Older adults with weaker muscle strength stand up from a sitting position with more dynamic trunk use. Sensors.

[ref-34] Wada Y, Shojima K, Tamaki K, Mori T, Kusunoki H, Onishi M, Tsuji S, Matsuzawa R, Nagai K, Sano K, Hashimoto K, Goto M, Nagasawa Y, Shinmura K (2023). Association between Timed Up-and-Go Test and future changes in the frailty status in a longitudinal study of Japanese community-dwelling older adults. Clinical Interventions in Aging.

[ref-35] Walz ID, Waibel S, Kuhner A, Gollhofer A, Maurer C (2023). Age-related changes in mobility assessments correlate with repetitive goal-directed arm-movement performance. BMC Geriatrics.

[ref-36] Watson W, Clapperton A, Mitchell R (2011). The burden of fall-related injury among older people in New South Wales. Australian and New Zealand Journal of Public Health.

[ref-37] Whitney SL, Wrisley DM, Marchetti GF, Gee MA, Redfern MS, Furman JM (2005). Clinical measurement of sit-to-stand performance in people with balance disorders: validity of data for the Five-Times-Sit-to-Stand Test. Physical Therapy.

