# Supplementary material for: Diagnostic accuracy of the Timed Up and Go and Five Times Sit-to-Stand tests for fall risk screening in community-dwelling older adults in northern Thailand: a retrospective study
Source: PeerJ. 2026 Apr 1;14:e21041. doi: 10.7717/peerj.21041 (PMC13050216; doi:10.7717/peerj.21041)
Supplement: Supplemental Information 1 [file peerj-14-21041-s001.pdf]

STROBE Statement—checklist of items that should be included in reports of observational studies

	Item No.	Recommendation	Page No.	Relevant text from manuscript
**Title and abstract**	1	(*a*) Indicate the study's design with a commonly used term in the title or the abstract	p.1	Title & Abstract: "Diagnostic accuracy of the Timed Up and Go and Five Times Sit-to-Stand tests …"
		(*b*) Provide in the abstract an informative and balanced summary of what was done and what was found		
**Introduction**				
Background/rationale	2	Explain the scientific background and rationale for the investigation being reported	p.2–4	Background: "Thailand is undergoing a significant demographic shift… Falls are associated with…"
Objectives	3	State specific objectives, including any prespecified hypotheses	p.4	Objectives: "This study aimed to assess and compare the diagnostic accuracy of the TUGT and the FTSST…"
**Methods**				
Study design	4	Present key elements of study design early in the paper	p.4	Study design: "This study employed a cross-sectional design using retrospectively collected data…"
Setting	5	Describe the setting, locations, and relevant dates, including periods of recruitment, exposure, follow-up, and data collection	p.4	Setting/Participants: "Community-dwelling adults aged 60 years and older in Rop Wiang Subdistrict…"
Participants	6	(*a*) *Cohort study*—Give the eligibility criteria, and the sources and methods of selection of participants. Describe methods of follow-up	p.4–5	Eligible adults ≥60 years in Rop Wiang Subdistrict;

		Case-control study—Give the eligibility criteria, and the sources and methods of case ascertainment and control selection. Give the rationale for the choice of cases and controls<br>*Cross-sectional study*—Give the eligibility criteria, and the sources and methods of selection of participants		inclusion/exclusion criteria and screening methods described.
		(*b*) *Cohort study*—For matched studies, give matching criteria and number of exposed and unexposed<br>*Case-control study*—For matched studies, give matching criteria and the number of controls per case		
Variables	7	Clearly define all outcomes, exposures, predictors, potential confounders, and effect modifiers. Give diagnostic criteria, if applicable	p.5-6	Fall-risk defined by fall history; exposures = TUGT, FTSST; confounders = age, sex, gait aid.
Data sources/ measurement	8*	For each variable of interest, give sources of data and details of methods of assessment (measurement). Describe comparability of assessment methods if there is more than one group	p.5-6	Data from EMR; assessments by licensed PTs using standardized FTSST and TUGT protocols.
Bias	9	Describe any efforts to address potential sources of bias	p.5	Retrospective data; standardized procedures and trained assessors minimized bias.
Study size	10	Explain how the study size was arrived at	p.5,7	All available eligible records; 113 participants included.

Continued on next page

Quantitative variables	11	Explain how quantitative variables were handled in the analyses. If applicable, describe which groupings were chosen and why	p.6.7	Continuous variables analyzed with t-test/Mann–Whitney; cut-offs for TUGT/FTSST from ROC & Youden's index; age groups 60–74 vs ≥75.
Statistical methods	12	(*a*) Describe all statistical methods, including those used to control for confounding	p.6-7	Logistic regression adjusting for age, sex, gait aid; ROC analysis for accuracy.
		(*b*) Describe any methods used to examine subgroups and interactions	p.8–9	Age-stratified subgroup analyses (60–74 vs ≥75 years).
		(*c*) Explain how missing data were addressed		
		(*d*) *Cohort study*—If applicable, explain how loss to follow-up was addressed<br>*Case-control study*—If applicable, explain how matching of cases and controls was addressed<br>*Cross-sectional study*—If applicable, describe analytical methods taking account of sampling strategy		
		(*e*) Describe any sensitivity analyses		
**Results**				
Participants	13*	(a) Report numbers of individuals at each stage of study—eg numbers potentially eligible, examined for eligibility, confirmed eligible, included in the study, completing follow-up, and analysed	p.7	113 older adults included; 61 fall-risk, 52 non-risk.
		(b) Give reasons for non-participation at each stage		
		(c) Consider use of a flow diagram		
Descriptive data	14*	(a) Give characteristics of study participants (eg demographic, clinical, social) and information on exposures and potential confounders	p.7, Table 1	Demographics, BMI, comorbidities, Thai-FRAT, gait aid, TUGT, FTSST.
		(b) Indicate number of participants with missing data for each variable of interest		
		(c) *Cohort study*—Summarise follow-up time (eg, average and total amount)	p.7-8, Tables 2–4	Logistic regression results, ROC AUC values, cut-off points.
Outcome data	15*	*Cohort study*—Report numbers of outcome events or summary measures over time		
		Case-control study—Report numbers in each exposure category, or summary measures of exposure		
		Cross-sectional study—Report numbers of outcome events or summary measures		

Main results	16	(*a*) Give unadjusted estimates and, if applicable, confounder-adjusted estimates and their precision (eg, 95% confidence interval). Make clear which confounders were adjusted for and why they were included	p.7-8	ORs with 95% CI for TUGT (1.16) and FTSST (1.11), adjusted for age, sex, gait aid.
		(*b*) Report category boundaries when continuous variables were categorized	p.8	Cut-off thresholds: TUGT 17.16 sec, FTSST 15.46 sec (overall); stratified by age.
		(*c*) If relevant, consider translating estimates of relative risk into absolute risk for a meaningful time period		

Continued on next page

Other analyses	17	Report other analyses done—eg analyses of subgroups and interactions, and sensitivity analyses	p.8, Table 3	Age-stratified subgroup analyses (60–74 vs ≥75 years). No sensitivity analyses.
**Discussion**				
Key results	18	Summarise key results with reference to study objectives	p.8-9	ROC showed TUGT (AUC 0.827) and FTSST (AUC 0.705) with age-specific cut-offs.
Limitations	19	Discuss limitations of the study, taking into account sources of potential bias or imprecision. Discuss both direction and magnitude of any potential bias	p.12	Single-region recruitment; retrospective data; reliance on fall history.
Interpretation	20	Give a cautious overall interpretation of results considering objectives, limitations, multiplicity of analyses, results from similar studies, and other relevant evidence	p.8–12	Findings consistent with prior studies; highlight complementary role of performance-based vs multifactorial tools.
Generalisability	21	Discuss the generalisability (external validity) of the study results	p.12	Limited to northern Thailand; generalizability restricted; need larger diverse samples.
**Other information**				
Funding	22	Give the source of funding and the role of the funders for the present study and, if applicable, for the original study on which the present article is based	p.13	Acknowledgements; no external funding stated, hospital staff support only.

*Give information separately for cases and controls in case-control studies and, if applicable, for exposed and unexposed groups in cohort and cross-sectional studies.

Note: An Explanation and Elaboration article discusses each checklist item and gives methodological background and published examples of transparent reporting. The STROBE checklist is best used in conjunction with this article (freely available on the Web sites of PLoS Medicine at http://www.plosmedicine.org/, Annals of Internal Medicine at http://www.annals.org/, and Epidemiology at http://www.epidem.com/). Information on the STROBE Initiative is available at www.strobe-statement.org.
